# From Vial to Vein: Crucial Gaps in Mesenchymal Stromal Cell Clinical Trial Reporting

**DOI:** 10.3389/fcell.2022.867426

**Published:** 2022-04-13

**Authors:** Danielle M. Wiese, Catherine A. Wood, Lorena R. Braid

**Affiliations:** ^1^ Aurora BioSolutions Inc., Medicine Hat, AB, Canada; ^2^ Simon Fraser University, Burnaby, BC, Canada

**Keywords:** mesenchymal stromal (stem) cell (MSC), ATMP, clinical trial, retrospective analysis, cell therapy (CT), regulatory approval, cell fitness, cell potency

## Abstract

Retrospective analysis of clinical trial outcomes is a vital exercise to facilitate efficient translation of cellular therapies. These analyses are particularly important for mesenchymal stem/stromal cell (MSC) products. The exquisite responsiveness of MSCs, which makes them attractive candidates for immunotherapies, is a double-edged sword; MSC clinical trials result in inconsistent outcomes that may correlate with underlying patient biology or procedural differences at trial sites. Here we review 45 North American MSC clinical trial results published between 2015 and 2021 to assess whether these reports provide sufficient information for retrospective analysis. Trial reports routinely specify the MSC tissue source, autologous or allogeneic origin and administration route. However, most methodological aspects related to cell preparation and handling immediately prior to administration are under-reported. Clinical trial reports inconsistently provide information about cryopreservation media composition, delivery vehicle, post-thaw time and storage until administration, duration of infusion, and pre-administration viability or potency assessments. In addition, there appears to be significant variability in how cell products are formulated, handled or assessed between trials. The apparent gaps in reporting, combined with high process variability, are not sufficient for retrospective analyses that could potentially identify optimal cell preparation and handling protocols that correlate with successful intra- and inter-trial outcomes. The substantial preclinical data demonstrating that cell handling affects MSC potency highlights the need for more comprehensive clinical trial reporting of MSC conditions from expansion through delivery to support development of globally standardized protocols to efficiently advance MSCs as commercial products.

## Introduction

Mesenchymal stromal cell (MSC) products are rapidly advancing as clinical treatments for a range of inflammatory diseases and regenerative medicine applications (Davies et al., 2017; Martin et al., 2019; Levy et al., 2020; Wright et al., 2021). MSC therapies have consistently proven safe (Levy et al., 2020; Krampera and le Blanc, 2021), but clinical outcomes from both autologous and allogeneic MSC trials have been variable and often less beneficial than in preclinical studies (Galipeau and Sensébé, 2018; Martin et al., 2019; Levy et al., 2020; Krampera and le Blanc, 2021). The inconsistent performance of MSC products has been attributed to numerous factors, most of which remain poorly understood or controlled. These have been comprehensively reviewed by others and include MSC heterogeneity between donors, tissues of origin and expansion level (Martin et al., 2019; le Blanc and Davies, 2018; Wiese et al., 2019a; Galipeau et al., 2021), preparation/manufacturing protocols (de Wolf et al., 2017; Mennan et al., 2019; Yin et al., 2019; Levy et al., 2020), administration route (Braid et al., 2018; Giri and Galipeau, 2020; Levy et al., 2020; Moll et al., 2020; Galipeau et al., 2021) and the underlying biological differences between patient recipients (Martin et al., 2019; Levy et al., 2020; Moll et al., 2020; Galipeau et al., 2021).

The realization of MSCs as advanced therapy medicinal products/advanced medicinal products (ATMP/AMP) requires global standardization of MSC manufacturing protocols, critical quality attributes, release criteria, and product preparation and delivery protocols at treatment sites (Mendicino et al., 2014; de Wolf et al., 2017; Viswanathan et al., 2019; Galipeau et al., 2021; Wilson et al., 2021; Wright et al., 2021). Retrospective analysis of clinical trial outcomes is a vital exercise to identify the practices that correlate with successful outcomes and those that result in variable outcomes or unsatisfactory efficacy. Statistically powered comparisons of trial procedures and outcomes are limited, however, by the degree to which clinical trial data are recorded and reported.

In this review, we analyze the product and procedural information provided in peer-reviewed clinical trial reports published since 2015. Our analysis focuses on reporting of cell handling procedures from dose preparation–either fresh or thawed–through completion of cell transfer. Surprisingly, we discovered that few clinical trials specify and/or report the handling of MSC products during this window in which the cells are vulnerable to insult and may experience uncontrolled conditions. This lack of information precludes retrospective analysis of the influence of product handling and delivery with clinical outcomes.

## Methods

### Search Strategy

The search terms *mesenchymal stromal cell clinical trial* and *mesenchymal stem cell clinical trial* were searched in PubMed and Google Scholar with filters to include the clinical trial article type, published from 2015 to 2021 inclusive, with an available abstract and full text. These queries returned 471 articles effective 21 January 2022.

### Report Selection and Data Extraction

The reports were filtered to include only trials using human-derived live MSC products for human use. Because reporting standards can vary by region, we further limited the scope of our analysis to clinical trials performed in North America. Rationale and Design articles were excluded. These refinements produced 45 peer-reviewed clinical trial reports for analysis.

Data was extracted verbatim from the curated reports according to four categories:

1) Trial and report particulars: Authors, article doi, trial location, publication year, trial phase, product name, affiliate company and clinical trial identifier
2) Study design: Disease or injury indication, administration route, MSC tissue of origin, selected MSC population (if any), MSC state (fresh, cryopreserved or culture-rescued after thaw) and donor relationship (allogeneic or autologous)
3) Dose preparation and handling: MSC dose (per kg and/or mean number), MSC concentration, delivery buffer, rate and duration of cell transfer, dose scheme, storage conditions and duration between dose preparation and administration, and miscellaneous handling details as listed

Where applicable: cryopreservation mode (aliquot or bag), cryomedia formulation, thaw procedures and cell recovery protocols.

4) MSC product characterization: culture media formulation, MSC population doubling level or passage, and quality control attributes including safety (sterility, endotoxin, *mycoplasma*, viral pathogens, karyotyping, residual FBS, tumorigenesis and others as listed), identity (morphology, surface marker profiles, multilineage potential, HLA profiling, clonogenicity and others as listed), functional attributes (PMBC suppression, cytokine expression, IDO-1 expression, T-cell proliferation, others as listed) and viability including post-thaw viability for cryopreserved products.

## Results

### Clinical Trial Parameters

The reports predominantly described Phase 1 clinical trials (44%) performed in the United States (90%). The therapeutic indication and clinical trial identifier associated with each publication are listed in Supplementary Table S1. The trials spanned a range of indications, including Graft versus Host Disease (GVHD), autoimmune diseases, cardiovascular injury and disease, sepsis, cancer and others (Supplementary Table S1). The majority of trials used bone marrow-derived (BM) MSCs (71%) delivered intravenously (IV; 40%).

All the clinical trial reports specified the MSC tissue of origin, whether the cell source was autologous or allogeneic, and the administration route (Supplementary Table S1; Tables 1, 2). Most of the trials (93%) reported the dose of MSCs in units of cells/kg patient weight, or mean cells per patient (Table 1). Three trials (7%) did not disclose or even quantify the number of cells per dose (Table 1). Twenty-three trials (51%) included dose-escalation schemes. Twenty-six trials (58%) used fixed doses rather than a dose/kg scheme (Table 1).

**TABLE 1:** Clinical trial publications inconsistently report details relevant to MSC dose preparation and bedside handling. Dashed lines represent unreported data.

Author	Administration Route	Cell dose		Cell delivery buffer	Rate and/or duration of administration	Dose and/or delivery detail	Prep-to-admin storage and timing
		# per kg	Mean #				
Amirdelfan et al. (2021)	Intradiscal	—	6 or 18 M	Hyaluronic acid (HA) carrier	—	2 ml (1 ml of 30 or 90 M cells/5 ml + 1 ml 1% HA)	Thawed and combined with HA carrier at time of administration
Lanzoni et al. (2021)	IV	—	100 ± 20 M	Plasma-Lyte, HSA, Heparin	10 ± 5 min	2 × 50 ml dose Plasma-Lyte, HSA, Heparin (D0, D3)	Thaw quickly, less than 3 h from thaw to administration
Bolli etal. (2018); Bolli et al. (2021)	Endocardial injections	—	75–150 M	Plasma-Lyte	—	6 ml	—
Soder et al. (2020)	IV	—	—	—	—	—	Thawed immediately on day of administration
Kurtzberg et al. (2020)	IV	2 M	50 M	Plasma-Lyte A	1 h	50 ml dose	Thawed and resuspended immediately before administration
Kebriaei et al. (2020)	IV	2 M	—	Plasma-Lyte, 50 g/L (5%) HSA, 10% DMSO	4–6 ml/min	—	Thawed and immediately infused
Chahal et al. (2019)	Intraarticular	—	1, 10 or 50 M	2.5% patient serum in Plasma-Lyte A	—	Dose in 6.5 ml+/- 1.5 ml	15–25°C for 8 h in Plasma-Lyte A then 2–10°C for 24 h
Schlosser et al. (2019)	IV	0.3, 1 or 3 M (total ≤300 M)	—	80% Plasma-Lyte A, 20% Alburex-25 human albumin	20 min (10 ml), 40 min (35 ml) or 60 min (100 ml) by dose cohort	—	—
Berry et al. (2019)	IT and IM injection (bicep and tricep)	—	125 M IT, 48 M IM	Culture media (DMEM)	—	5 ml IT and 1 ml × 24 IM; DMEM placebo	Validated shipping system at controlled temperature 2–8°C
Dozois et al. (2019)	Fistula plug	—	20 M/plug	Maintained in Lactated Ringer’s solution until delivery	—	—	—
Yau et al. (2019)	Intramyocardial	—	150 M	Cryoprotective medium as sham	15 min	16–20 injections of 0.2 ml	Thawed longer than 90 min discarded
Levy et al. (2019)	IV	0.5, 1, or 1.5 M	—	Lactated Ringer’s solution	2 ml/min	1 M cells/ml in 1–3 × 60 ml syringes; 0.1 ml intradermal for patient reactivity prior	Stored at 2 to 8°C and infused within 8 h
Singer et al. (2019)	IT	—	10 M, 2 × 50 M or 2 × 100 M	Lactated Ringer’s solution	1–2 min	Dose followed by 1 ml flush	Used within 12 h of preparation
Myerson et al. (2019)	Arthrodesis surgery	N/A (device)	—	—	—	—	—
Schweizer et al. (2019)	IV	1 M or 2 M (max 100 M or 200 M total)	—	6% hetastarch in 0.9% NaCl injection, 2% HSA, 5% DMSO	—	—	—
Powell and Silvestri (2019)	Intratracheal	10 M (2 ml/kg in 2 aliquots) or 20 M (4 ml/kg in 4 aliquots)	—	Normal saline	5–10 min	5 M/ml	Administered within 3 h of thawing and resuspension
Chan et al. (2020)	Intramyocardial	—	Targeted 150 M, minimum 15 M	0.9% NaCl	—	3 ml in 30 × 100 µl	—
Harris et al. (2018)	IT	—	5.3–10 M (3 doses 3 months apart)	Saline	—	—	—
McIntyre et al. (2018)	IV	0.3, 1 or 3 M to max of 300 M	—	80% Plasma-Lyte A, 20% Alburex-25 human albumin	20 min (10 ml), 40 min (35 ml) or 60 min (100 ml) by dose cohort	—	—
Matthay et al. (2019)	IV	10 M	—	Plasma-Lyte A	60–80 min	100 ml dose	—
Swaminathan et al. (2018)	Intraaortic	2 M	—	10% DMSO, 5% HSA in Plasma-Lyte A, pH 7.4^a^	1–3 min	100 ml dose	On refrigerated gel packs and administration within 8 h preparation
Keller et al. (2018)	IV	1, 2 or 4 M	5 M	Plasma-Lyte, 0.5% DMSO	2–3 ml/min during the first 15 min, with the option to be adjusted up to 5 ml/min if tolerated	Cells diluted 5-fold in 100 ml	—
Tompkins et al. (2017)	IV	—	100 or 200 M	0.9% saline^a^	2 ml/min	100 ml; squeeze infusion bag every 15 min, 25 ml flush at end	—
Glassberg et al. (2017)	IV	—	20, 100 or 200 M	PBS, 1% HSA^a^	—	—	Cryo: thaw in 37°C water bath, wash, resuspended; Fresh: resuspended^a^
Dietz et al. (2017)	Fistula plug	—	20 M per plug	Lactated Ringer’s solution	—	—	—
Golpanian et al. (2017)	IV	—	20, 100 or 20 M	0.9% saline^a^	2 ml/min	100 ml; squeeze infusion bag every 15 min, 25 ml flush at end	—
Florea et al. (2017)	Transendocardial	—	20 or 100 M	PBS +1% HSA or Plasma-Lyte A+ 1% HSA^a^	—	20 M/ml; 0.5 cc per injection × 10	Thaw at 37°C in water bath, pellet resuspended^a^
Saad et al. (2017)	Intraarterial	0.1 or 0.25 M	—	Lactated Ringer’s solution	5 min	10 ml	—
Butler et al. (2017)	IV	1.5 M	—	Lactated Ringer’s solution	—	1M/ml, 1 ml/kg	Thawed within pharmacy, infusion within 8 h
Bajestan et al. (2017)	Alveolar graft	—	15–44 M/ml, 2–5 ml/patient	Isolyte +0.5% HSA mixed with b-TCP carrier	—	10 ml ixmyelocel-t in Isolyte +0.5% HSA mixed with b-TCP carrier; 2.5 ml/patient	At 4°C for up to 40 h
Hare et al. (2017)	Transendocardial	—	100 M (≥80 M autologous)	PBS +1% HSA or Plasma-Lyte A+ 1% HSA^a^	0.4 ml/min, 10 × 0.5 ml each	20 M/ml	Thaw at 37°C in water bath, pellet resuspended^a^
Harris et al. (2016)	IT	—	—	Saline with CSF	—	Saline with 3 ml CSF then 2 ml CSF flush	—
Steinberg et al. (2016)	Post-craniostomy implant	—	2.5, 5 or 10 M	—	10 µl per minute, 15 min per track × 3 tracks	—	—
Dhere et al. (2016)	IV	2, 5 or 10 M	—	Plasma-Lyte A with 0.05% HSA	Roughly 60 min	4 M cells/ml	—
Staff et al. (2016)	IT	—	10, 50, 50 M × 2, 100 M	Lactated Ringer's solution	1‐2 min	2 or 10 ml	Administered post-thaw or post-thaw + 4 days
Castillo-Cardiel et al. (2017)	To mandibular fracture line pre-open reduction and internal fixation (ORIF)	—	10–600 M from 50cc adipose tissue	—	—	—	—
Coetzee et al. (2016)	Arthrodesis surgery	N/A (device)	—	—	—	—	—
Patel et al. (2016)	Transendocardial	—	35–295 M^a^	—	—	5.8–8.4 ml was delivered as a series of 12–17 injections of 0.4 ml each^a^	—
Levy et al. (2016)	Corpora cavernosum base injection	—	1 ml product (# not quantified)	Isotonic saline	—	1.5 ml of 3 ml dilution	—
Perin et al. (2015)	Transendocardial	—	25, 75 or 150 M	Cryoprotective medium as sham^a^	—	16–20 injections of 0.2 ml	—
Levy et al. (2015)	Peyronie plaques, corpora injection	—	—	Isotonic saline	—	Up to 2 ml of 3 ml dilution	—
Skyler et al. (2015)	IV	0.3, 1 or 2 M	—	Normal saline	45 min	100 ml	Thawed immediately before use
Wilson et al. (2015)	IV	1, 5 or 10 M	—	Plasma-Lyte A	60–80 min	100 ml	2 h of stability, then 60–80 min gravity feed
Maziarz et al. (2015)	IV	1, 5 or 10 M (repeat 1 or 5M × 3/week or 5M × 5/week	—	Plasma-Lyte A, 5% DMSO	5–10 ml/min	23–61 ml or 100–143 ml or 133–294 ml (diluted based on body weight)	Infused within 6 h after thaw
Pettine et al. (2015)	Intradiscal	—	∼726 M (121 ± 11 M/ml × 6)	Non-expanded BM concentrate	—	6 ml	—

a Denotes publications which have information referenced in external references or supplemental material. Abbreviations: BM, bone marrow; D, day; DMSO, dimethylsulfoxide; FBS, fetal bovine serum; HSA, human serum albumin; IM, intramuscular; IT, intrathecal; IV, intravenous; M, million; MEM, modified eagle’s media; min, minute; N/A, not applicable; NaCl, sodium chloride; NEAA, non-essential amino acids; P, passage; PBS, phosphate buffered saline; PDL, population doubling level.

**TABLE 2:** Clinical trial publications underreport MSC manufacturing details. Dashed lines represent unreported data.

Author	Donor	Manufacturing information		Other preparation details	MSC state	Cryopreservation mode	Cryomedia formulation
		Culture media	MSC culture age				
Amirdelfan et al. (2021)	Allogeneic	—	—	—	Frozen	—	—
Lanzoni et al. (2021)	Allogeneic	DMEM Low Glucose, 10% platelet gold, 1 × GlutaMAX, 1 × MEM-NEAA	—	—	Frozen	—	—
Bolli etal. (2018); Bolli et al. (2021)	Autologous	Lymphocyte cell separation media	—	—	Frozen	—	—
Soder et al. (2020)	Allogeneic	—	P5	—	Frozen	Aliquot	Plasma-Lyte A, DMSO, HSA
Kurtzberg et al. (2020)	Allogeneic	—	P5	—	Frozen	Aliquot	Plasma-Lyte A, DMSO, HSA
Kebriaei et al. (2020)	Allogeneic	Supplemented with 10% FBS^a^	P5	—	Frozen	Bag	Plasma-Lyte, 50 g/L (5%) HSA, 10% DMSO
Chahal et al. (2019)	Autologous	DMEM low glucose, 1% Glutamax, 10% FBS	P3 (day 30) or P4 (day 37)	Washed 2x in Plasma-Lyte A, 1x in Plasma-Lyte A+ 2.5% patient serum (excipient)	Fresh	N/A	—
Schlosser et al. (2019)	Allogeneic	NutriStem XF	PDL ≤12	Culture 5–12 days after thaw (PDL≤18)	Culture-rescued after thaw	—	—
Berry et al. (2019)	Autologous	—	—	3–4 weeks culture for neurotrophic factor secretion	Fresh	N/A	10% DMSO in growth medium, controlled rate, pre-MSC-NTF generation^a^
Dozois et al. (2019)	Autologous	—	—	Thawed to adhere to fistula plug (proprietary)	Frozen	—	—
Yau et al. (2019)	Allogeneic	—	—	—	Frozen	Aliquot4 × 1 ml	7.5% DMSO, 50% *α*-MEM, 42.5% ProFreeze^a^
Levy et al. (2019)	Allogeneic	—	P4	5% O2; washed in Lactate Ringer’s solution	Frozen	Aliquot	Cryostor CS10
Singer et al. (2019)	Autologous	—	—	Thaw from cryo, culture in PLTMax for 3–5 days	Culture-rescued after thaw	—	—
Myerson et al. (2019)	Allogeneic	—	—	—	—	—	—
Schweizer et al. (2019)	Allogeneic	α-MEM, 2 mM l‐glutamine, 10% FBS, no antibiotics	—	—	Frozen	Bag20 ml	6% hetastarch in 0.9% NaCl injection, 2% HSA, 5% DMSO
Powell and Silvestri (2019)	Allogeneic	—	—	—	Frozen	—	—
Chan et al. (2020)	Autologous	α-MEM, 20% FBS, gentamicin	To P3 in 21 days	N/A	Fresh	N/A	N/A
Harris et al. (2018)	Autologous	Lonza NPMM	2–3 weeks after thaw at P2-3		Culture-rescued after thaw	—	—
McIntyre et al. (2018)	Allogeneic	NutriStem XF	PDL ≤12	Culture 5–12 days after thaw (PDL≤18)	Culture-rescued after thaw	—	—
Matthay et al. (2019)	Allogeneic	—	—	Wash to remove DMSO before resuspension	Frozen	Aliquot	Contains DMSO
Swaminathan et al. (2018)	Allogeneic	—	—	—	Frozen	Bag20 ml	20 ml (120 M cells) PlasmaLyte A w/10% DMSO, 5% HSA, pH 7.4^a^
Keller et al. (2018)	Allogeneic	α-MEM, 9.8% HyClone Characterized FBS	—	—	Frozen	—	20 ml, 2.5% DMSO
Tompkins et al. (2017)	Allogeneic	α-MEM, 20% FBS	P1 (21–24 days)^a^	Wash with Plasma-Lyte A+ 1% HSA^a^	Fresh	N/A	N/A
Glassberg et al. (2017)	Allogeneic	α-MEM, 20% FBS	P1 (21–24 days)^a^	Washed^a^	Fresh and frozen	—	Pentaspan (10% pentastarch in 0.9% NaCl), 2% HSA, 5% DMSO^a^
Dietz et al. (2017)	Autologous	—	—	Thaw from cryo, bioreactor 3–6 days for plug adherence	Culture-rescued after thaw	—	—
Golpanian et al. (2017)	Allogeneic	α-MEM, 20% FBS	P1 (21–24 days)^a^	Wash with Plasma-Lyte A+ 1% HSA^a^	Fresh	N/A	N/A
Florea et al. (2017)	Allogeneic	α-MEM, 20% FBS	P1 (21–24 days)^a^	—	Frozen	—	Pentaspan (10% pentastarch in 0.9% NaCl), 2% HSA, 5% DMSO^a^
Saad et al. (2017)	Autologous	Isolated 6 weeks prior, 2 weeks in Advanced MEM with PLTMax (5% platelet lysate, 100 U/ml penicillin, 100 g/ml streptomycin, 2 mM l-glutamine)	—	N/A	Fresh	N/A	N/A
Butler et al. (2017)	Allogeneic	—	—	Hypoxia	Frozen	—	Cryostor CS10
Bajestan et al. (2017)	Autologous	IMDM, 10% FBS, 10% horse serum, 5 mM hydrocortisone	12 days in bioreactor	N/A	Fresh	N/A	N/A
Hare et al. (2017)	Autologous and Allogeneic	α-MEM, 20% FBS	P1 (21–24 days)^a^	—	Frozen	—	Pentaspan (10% pentastarch in 0.9% NaCl), 2% HSA, 5% DMSO^a^
Harris et al. (2016)	Autologous	2–3 passages/7–54 days in Lonza MSCGM +10% patient serum, plus 7–24 days in Lonza NPMM		N/A	Fresh	N/A	N/A
Steinberg et al. (2016)	Allogeneic	—	—	—	—	—	—
Dhere et al. (2016)	Autologous	α-MEM, 10% HSA	P1	N/A	Fresh	N/A	N/A
Staff et al. (2016)	Autologous	Advanced MEM, 5% hPL	<P5	—	Frozen	Aliquot	—
Castillo-Cardiel et al. (2017)	Autologous	DMEM, 10% FBS, antibiotics	24 h	N/A	Fresh	N/A	N/A
Coetzee et al. (2016)	Allogeneic	—	—	—	—	—	—
Patel et al. (2016)	Autologous	—	12 days in bioreactor	N/A	Fresh	N/A	N/A
Levy et al. (2016)	Allogeneic	—	—	—	—	—	—
Perin et al. (2015)	Allogeneic	—	P5 or <20 PDL	—	Frozen	Aliquot4 × 1 ml	4% DMSO, 50% *α*-MEM, 42.5% ProFreeze
Levy et al. (2015)	Allogeneic	—	—	—	—	—	—
Skyler et al. (2015)	Allogeneic	Media (unspecified), FBS	—	—	Frozen	—	4% DMSO, 50% *α*-MEM, 42.5% ProFreeze
Wilson et al. (2015)	Allogeneic	—	—		Frozen	—	Contains DMSO
Maziarz et al. (2015)	Allogeneic	FBS	—	Wash in HSA before cryo	Frozen	—	Contains DMSO
Pettine et al. (2015)	Autologous	—	—	—	—	—	—

a Denotes publications which have information referenced in external references or supplemental material. Abbreviations: cryo, cryopreservation; DMSO, dimethylsulfoxide; FBS, fetal bovine serum; HSA, human serum albumin; IM, intramuscular; IV, intravenous; M, million; MEM, modified eagle medium (D, Dulbecco’s); MSC, mesenchymal stromal cell; N/A, not applicable; NaCl, sodium chloride; NEAA, non-essential amino acids; NTF, neurotrophic factor-secreting; P, passage; PDL, population doubling level.

### Reported MSC Product Characterization

Some form of cell product characterization was usually reported (89%), although the assessment criteria used was mixed (Supplementary Table S2). Viability was the most commonly reported metric, but the acceptable threshold ranged from 50 to 98% between trials (Supplementary Table S2). Studies using frozen cells stipulate whether viability assessments were made before cryopreservation, on a sample thawed lot, or per vial/bag at the time of use. Safety criteria, including tests for bacterial, fungal and viral contamination, chromosomal stability and residual FBS, were reported in 32 studies (71%; Supplementary Table S2). Thirty-three reports (73%) listed cell identity tests, including surface marker profiling, multi-lineage differentiation, and clonogenicity (Supplementary Table S2). Functional assessments were only reported for 12 clinical trials (27%) and included peripheral blood mononuclear cell (PBMC) and T-cell suppression, IDO-1 expression after IFN-γ stimulation, or secretion of other relevant proteins (Supplementary Table S2).

Details related to product formulation and handling were poorly documented. Twenty-five publications (55%) failed to fully define the medium in which the MSCs were expanded or administered, and 23 reports (51%) provided no information about the population doubling level (culture age) of the cells (Table 2). Of the 21 reports (47%) that provided some description of MSC expansion level, 10 (22%) only provided number of days in culture. Three (7%) reports provided discrete population doubling levels; the remaining studies reported passage number.

### Reported MSC Product Handling

Most trials (62%) used previously frozen MSCs, while six publications (13%) did not stipulate whether their MSC products were derived from fresh cultures or had been thawed (Table 2). Of the 28 publications that used previously frozen MSC products, nearly half did not list the cryopreservation media (Table 2). Cryo-rescue procedures were essentially unreported, even though all but four trials administered MSCs directly following thaw without a recovery period or transfer of cells from cryopreservation media to delivery buffer/vehicle. Only seven papers stated that a wash step was performed, but no further details of the wash procedures were provided (Table 1).

Injection/infusion buffers were fairly well reported (91%) and predominately consisted of Plasma-Lyte, Plasma-Lyte A, Lactated Ringer’s solution, and saline with or without human serum albumin (HSA) or dimethylsulfoxide (DMSO) at varying concentrations (Table 1). Buffer solution was not used in an AD MSC bone allograft device in arthrodesis surgery (Coetzee et al., 2016; Myerson et al., 2019). One publication reported intradiscal injection of non-expanded BM concentrate (Pettine et al., 2015).

Duration of cell transfer was reported for the majority (78%) of trials that used IV infusion, either in minutes or ml/min (Table 1). Infusion time ranged from 5 min to 1 h. Of the trials using other administration routes, 28% reported the duration or rate of administration (Table 1). Most reports (84%) provided no information about the elapsed time from when the dose was prepared until cell transfer was complete (Table 1). Seven (16%) reports specified a maximum elapsed time from dose prep or thaw to administration, which ranged from 90 min to 12 h (Table 1). The three studies that included product handling protocols each used different methods; prepared doses were held in refrigeration, on cold packs or at room temperature (Table 1).

## Discussion

MSCs are fundamentally responsive to subtle changes in their environment. MSCs respond to changes in atmospheric gases (Lin et al., 2014; Gorgun et al., 2021; Roemeling-Van Rhijn et al., 2013; Ejtehadifar et al., 2015; Kang et al., 2019; von Bahr et al., 2019), temperature (Stolzing et al., 2006; Kubrova et al., 2020; Shimoni et al., 2020), hydrostatic pressure (Steward et al., 2012; Becquart et al., 2016; Pattappa et al., 2019) and aggregation (Robb et al., 2019; Yuan et al., 2019; Burand et al., 2020; Xie et al., 2021). It is surprising then, that the steps and duration between dose preparation and delivery of MSC therapies are ill-defined and under-reported. We predict that bedside handling of MSC products may contribute substantially to the variability and reduced efficacy documented in clinical trials. Retrospective analysis to test this hypothesis, however, is currently impossible due to the absence of relevant information (Sart et al., 2014).

As example, MSCs have a natural tendency to self-assemble and form aggregates [reviewed in (Myerson et al., 2019)]. It has been reported that spontaneous aggregation can alter the immunosuppressive properties of MSCs, rendering them incapable of T cell suppression (Lanzoni et al, 2021). Thus, steps must be taken to control MSC aggregation between dose preparation and the completion of cell transfer. Even though cell doses were held for up to 12 h in the reviewed clinical trials, almost no measures to manage cell aggregation were described. Two studies reported squeezing the bag every 15 min during infusion, but no other reports described strategies to mitigate spontaneous aggregation. If the reports had documented the steps taken (if any) to prevent MSC aggregation during administration, retrospective analysis could potentially reveal whether implementing these strategies improves clinical outcomes.

Retrospective analysis could similarly be used to determine whether wash number, wash duration, centrifugation speed and buffer composition correlates with clinical outcomes. Thawed cells are fragile so thaw temperatures, duration and subsequent wash steps likely impact MSC fitness. The steps used to reconstitute frozen MSCs thawed immediately prior to administration were never reported. Moreover, few trials that thawed frozen MSCs immediately prior to administration stated the density at which the cells were cryopreserved, composition of the cryopreservation media, how the cells were thawed, whether or not they were washed, frequency of washing and the wash buffer used.

Currently, any changes in MSC fitness and performance in the hours between dose preparation and completion of infusion or injection is a black box devoid of data. To our knowledge, few studies have formally tested potential loss of function through sampling of MSC products during this window, or by recapitulating these conditions in laboratory tests (Pal et al., 2008; Chen et al., 2013; Niu et al., 2013). Intermittent bedside product testing admittedly is a logistical challenge. Thus, we suggest that clinical trial design include laboratory development of defined bedside procedures to ensure that the patient receives the same quality of MSC product that was prepared earlier and was subject to quality testing. Establishing and reporting these cell handling procedures, as well as any deviations from these protocols, may provide invaluable insight for retrospective analysis and ultimately ensure that patients consistently receive high quality MSC treatments.

There is a global movement towards standardization of MSC products. Such standardization includes development of tests to establish minimum cell performance criteria (Chinnadurai et al., 2018; Galipeau and Sensébé, 2018; Wiese et al., 2019b; Martin et al., 2019; Wiese and Braid, 2020a; Wiese and Braid, 2020b; Moll et al., 2020; Galipeau et al., 2021; Krampera and le Blanc, 2021), which are a critical to obtain regulatory approval for commercialization (Mendicino et al., 2014; Galipeau et al., 2015; de Wolf et al., 2017; Galipeau and Sensébé, 2018). Consistent with this movement, we found that most clinical trials reported some type of cell characterization. Viability and cell identity, based on accepted MSC cell surface profiles, were the most commonly reported tests. Consistent with a recent review of MSC characterization in clinical trials (Wilson et al., 2021), cell performance in functional assays or surrogate potency assays was documented infrequently, and performance thresholds were not disclosed. Post-thaw viability was also reported far less frequently than expected, especially since most of the trials used cryo-rescued cells.

We propose that ongoing global efforts to define the critical quality attributes of MSC ATMPs and subsequent release criteria be mindful of the need to identify markers and tests that can rapidly report MSC fitness and potency. These rapid-response markers will enable future development of in-process and bedside testing of MSC products, an important advancement in the realization of MSCs as commercially viable cell therapies.

Finally, retrospective analysis would be better enabled by establishing formal guidelines for clinical trial reporting. A recent clinical trial design by Baker et al*.* (2021) provides an excellent model to establish reproducible and transparent bedside cell handling procedures. We propose that clinical trial reports include all available cell characterization data and carefully document bedside handling of MSC products. Making this information readily available in the main report rather than citing other publications would facilitate accessibility for statistical analysis of large data sets and improve confidence that the data correlates with actual events and cell doses used in the trial.

## Conclusion

We urge the MSC community to incorporate and report bedside MSC handling protocols and best practices in clinical trial design and reporting. The notable lack of information and data surrounding how these exquisitely responsive cells are treated when the cells are most vulnerable is not likely an issue of propriety. Rather, this aspect of the cell therapy journey from vial to vein appears to have been designated as arbitrary, a classification that we argue is flawed. Documenting and reporting bedside cell processing and handling procedures will aid effective retrospective analysis of clinical trial outcomes and expedite the commercialization of MSC products.

## Abbreviations

CFU, colony forming units; cryo, cryopreservation; ELISA, enzyme linked immunosorbent assay; FBS, fetal bovine serum; h, hour; HLA, human leukocyte antigen; IDO, indoleamine 2,3-deoxygenase; IFN, interferon; IL, interleukin; NTF, neurotrophic factor; PBMC, peripheral blood mononuclear cells; PCR, polymerase chain reaction; QC, quality control; TNF, tumor necrosis factor.
