# Supplementary material for: From Vial to Vein: Crucial Gaps in Mesenchymal Stromal Cell Clinical Trial Reporting
Source: Front Cell Dev Biol. 2022 Apr 13;10:867426. doi: 10.3389/fcell.2022.867426 (PMC9043315; doi:10.3389/fcell.2022.867426)
Supplement: Supplementary file 1 [file Table1.docx]

Supplementary Table 1. Clinical trial publications (n=45).

Shaded boxes represent unreported data.* denotes publications which have information referenced in external references or supplemental material. AD, adipose-derived; BM, bone marrow-derived; LVAD, left ventricular assist device; M, million; MSC, mesenchymal stromal cell; N/A, not applicable; NTF, neurotrophic factor-secreting; PL, placental-derived; UC, umbilical cord-derived.

| **Author** | **Location** | **Publication**  **Year** | **Trial Phase** | **Product Name**  **(Affiliate Company)** | **Identifier** | **Indication** | **Selected MSC Population** | **Tissue of Origin** |
| --- | --- | --- | --- | --- | --- | --- | --- | --- |
| Amirdelfan et al. (19) | USA | 2021 | 1b/2a | (MesoBlast, Ltd.) | NCT01290367 | Chronic low back pain)/degenerative disc disease | Mesenchymal precursor cell (MPC): STRO-3 immunoselected BM mononuclear cells (MNC) | BM |
| Lanzoni et al. (20) | USA | 2021 | 1/2a |  | NCT04355728 | COVID-19 | N/A | UC |
| Bolli et al. (21,22) | USA | 2021 | 2 | CCTRN CONCERT-HF | NCT02501811 | Ischaemic heart failure | BM MSC +/- cardiac progenitor/stem cells | BM |
| Soder et al. (23) | USA | 2020 | 1 |  | NCT03158896 | High risk or steroid refractory acute Graft versus host disease (aGVHD) | N/A | UC |
| Kurtzberg et al. (24) | USA | 2020 | 3 | Remestemcel-L (MesoBlast, Ltd.) | NCT04371393 | Paediatric refractory aGVHD | N/A | BM |
| Kebriaei et al. (25) | Multinational incl. Canada, USA | 2019 | 3 | Remestemcel-L (MesoBlast, Ltd.) | NCT04371393 | Refractory aGvHD | N/A | BM |
| Chahal et al. (26) | Canada | 2019 | 1/2a |  | NCT02351011 | Knee osteoarthritis | N/A | BM |
| Schlosser et al. (27) | Canada | 2019 | 1 |  | NCT02421484 | Septic shock | N/A | BM |
| Berry et al. (28) | USA | 2019 | 2 | NurOwn,  MSC-NTF | NCT02017912 | Amytrophic lateral sclerosis | N/A | BM |
| Dozois et al. (29) | USA | 2019 | 1 |  |  | Transsphincteric cryptoglandular fistulas | N/A | AD |
| Yau et al. (30) | Canada, USA | 2019 | 2 | (MesoBlast, Ltd.) | NCT02362646 | LVAD weaning post-myocardial infarction | MPC (STRO-3 immunoselected BM MNC) | BM |
| Levy et al. (31) | USA | 2019 | 1/2 | (Stemedica Cell Technologies, Inc) | NCT01297413 | Stroke | N/A | BM |
| Singer et al. (32) | USA | 2019 | 1/2 |  | NCT02315027 | Multiple system atrophy | N/A | AD |
| Myerson et al. (33) | USA | 2019 | (device) | AlloStem Cellular Bone Allograft (AlloSource) | NCT01413061 | Subtalar arthrodesis | AD MSC bone matrix | AD |
| Schweizer et al. (34) | USA | 2019 | 1 |  | NCT01983709 | Prostate cancer | N/A | BM |
| Powell et al. (35) | USA | 2019 | 1 |  | NCT02381366 | Extremely-low-birth-weight premature infants at high risk for bronchopulmonary dysplasia | N/A | UCB |
| Chan et al. (36) | USA | 2019 | 1 |  | NCT01557543 | Ischemic heart disease post-cardiac surgical revascularization | N/A | BM |
| Harris et al. (37) | USA | 2018 | 1 |  | NCT01933802 | Multiple sclerosis | MSC-NP (neural progenitors derived from BM MSC) | BM |
| McIntyre et al. (38) | Canada | 2018 | 1 |  | NCT02421484 | Septic shock | N/A | BM |
| Matthay et al. (39) | USA | 2018 | 2 |  | NCT02097641 | Acute Respiratory Distress Syndrome | N/A | BM |
| Swaminathan et al. (40) | Canada, USA | 2018 | 2 | AC607  (AlloCure Inc.) | NCT01602328 | Acute kidney surgery after cardiac surgery | N/A | BM |
| Keller et al. (41) | USA | 2018 | 1 |  | NCT02181712 | Bronchiolitis obliterans syndrome | N/A | BM |
| Tompkins et al. (42) | USA | 2017 | 2 |  | NCT02065245 | Aging frailty | BM MSC (from BM MNCs without characterization) | BM |
| Glassberg et al. (43) | USA | 2017 | 1 |  | NCT02013700 | Idiopathic pulmonary fibrosis | N/A | BM |
| Dietz et al. (44) | USA | 2017 | 1 |  | NCT01915927 | Crohn's disease perianal fistulas | N/A | AD |
| Golpanian et al. (45) | USA | 2017 | 1 |  | NCT02065245 | Aging frailty | N/A | BM |
| Florea et al. (46) | USA | 2017 | 2 |  | NCT02013674 | Ischemic cardiomyopathy | N/A | BM |
| Saad et al. (47) | USA | 2017 | 1/2A |  | NCT02266394 | Renovascular disease | N/A | AD |
| Butler et al. (48) | USA | 2017 | 2A |  | NCT02467387 | Nonischemic cardiomyopathy | N/A | BM |
| Bajestan et al. (49) | USA | 2017 | 1/2 | Ixmyelocel-t | Michigan Institute for Clinical Health Research (MICHR) Clinical Trials Pilot Program | Alveolar defects | BM MSC, monocytes/macrophages | BM |
| Hare et al. (50) | USA | 2017 | 1/2 |  | NCT01392625 | Ischemic cardiomyopathy | N/A | BM |
| Harris et al. (51) | USA | 2016 | 1 |  |  | Multiple sclerosis | MSC-NP (neural progenitors derived from BM MSC) | BM |
| Steinberg et al. (52) | USA | 2016 | 1/2a |  | NCT01287936 | Stroke | BM MSC (SB623, Notch-1 transient plasmid) | BM |
| Dhere et al. (53) | USA | 2016 | 1 |  | NCT01659762 | Crohn's disease | N/A | BM |
| Staff et al. (54) | USA | 2016 | 1 |  | NCT01609283 | Amyotrophic Lateral Sclerosis | N/A | AD |
| Castillo-Cardiel et al. (55) | Mexico | 2016 | 3 |  | NCT02755922 | Mandibular fracture | N/A | AD |
| Coetzee et al. (56) | USA | 2016 | 3 | AlloStem Cellular Bone Allograft (AlloSource) |  | Subtalar arthrodesis | AD MSC (combined with demineralized allograft bone in strip) | AD |
| Patel et al. (57) | USA | 2016 | 2B | Ixmyelocel-t | NCT01670981 | Congestive heart failure due to ischaemic dilated cardiomyopathy | BM MSC, monocytes/macrophages | BM |
| Levy et al. (58) | USA | 2016 | 1 |  | NCT02398370 | Erectile dysfunction | N/A | PL |
| Perin et al. (59) | USA | 2015 | 2 | (MesoBlast, Ltd.) | NCT00721045 | Heart failure | MPC (STRO-3 immunoselected BM MNC) | BM |
| Levy et al. (60) | USA | 2015 | 1 |  | NCT02395029 | Peyronie disease | N/A | PL |
| Skyler et al. (61) | USA | 2015 | 1/2 | Rexlemestrocel-L (MesoBlast, Ltd.) | NCT01576328 | Type 2 diabetes | MPC (STRO-3 immunoselected BM MNC) | BM |
| Wilson et al. (62) | USA | 2015 | 1 |  | NCT01775774 | Acute respiratory distress syndrome | N/A | BM |
| Maziarz et al. (63) | USA | 2015 | 1 | MultiStem (Athersys, Inc.) |  | GvHD (prophylaxis after hematopoietic cell transplantation) | BM multipotent adult progenitor cells (hypoxia, GFs, confluence) | BM |
| Pettine et al. (64) | USA | 2015 | 1 | Remestemcel-L (MesoBlast, Ltd.) |  | Discogenic low back pain | BM concentrated cells incl. MSC, non-expanded | BM |
