# Supplementary material for: From Vial to Vein: Crucial Gaps in Mesenchymal Stromal Cell Clinical Trial Reporting
Source: Front Cell Dev Biol. 2022 Apr 13;10:867426. doi: 10.3389/fcell.2022.867426 (PMC9043315; doi:10.3389/fcell.2022.867426)
Supplement: Supplementary file 2 [file Table2.docx]

Table S2: Reported safety, MSC identity, potency and viability assessments.

* denotes publications which have information referenced in external references or supplemental material.

Abbreviations: CFU, colony forming units; cryo, cryopreservation; ELISA, enzyme linked immunosorbent assay; FBS, fetal bovine serum; h, hour; HLA, human leukocyte antigen; IDO, indoleamine 2,3-deoxygenase; IFN, interferon; IL, interleukin; NTF, neurotrophic factor; PBMC, peripheral blood mononuclear cells; PCR, polymerase chain reaction; TNF, tumor necrosis factor; QC, quality control.

| **Author** | **Safety** | **MSC Identity** | **MSC Function** | **Viability** | **Post-thaw viability** |
| --- | --- | --- | --- | --- | --- |
| Amirdelfan et al. (19) | Sterility, endotoxins, and mycoplasma | Clonogenicity, surface marker profiling |  | Yes |  |
| Lanzoni et al. (20) | Endotoxin, Gram stain and 14 day sterility | CD90+, CD105+ >95%, CD34+, CD45+ <5% |  | Yes  (Trypan blue) | ≥ 80% |
| Bolli et al.  (21, 22) | Gram stain, sterility testing and endotoxins |  |  | Yes  (Trypan blue) | ≥ 70% |
| Soder et al. (23) | Sterility, endotoxins, mycoplasma, and chromosomal stability | Surface marker profiling | PBMC suppression | Yes  (Trypan blue) | ≥ 80% |
| Kurtzberg et al. (24) |  | Surface marker profiling incl. CD105+, CD156+, CD45-, TNFR1+ | Suppression of IL-2Rα expression on activated lymphocytes |  |  |
| Kebriaei et al.  (25) | Viral pathogens, mycoplasma, sterility, endotoxin, purity* | Positive for 10 surface antigens, negative for 3 hematopoietic markers* | Expression level of TNFR1, inhibition  of IL-2Rα expression on activated T cells* | Yes  (Trypan blue)* | ≥ 70%* |
| Chahal et al.  (26) | Endotoxin, Gram stain, mycoplasma* |  | Immunophenotying, differentiation, potency IFN-γ and TNF-α and gene expression* | Yes | >70% |
| Schlosser et al.  (27) | Sterility, adventitious agents, mycoplasma and genotyping stability | Surface marker profiling | IDO-1 expression and T cell suppression | Yes  (Trypan blue) | >80% |
| Berry et al. (28) | Sterility, mycoplasma, and endotoxin | Surface marker profiling | ELISA for NTF secretion | Yes |  |
| Dozois et al. (29) | Sterility (aerobic and anaerobic), mycoplasma, and cytogenetic analysis | Surface marker profiling, morphology, plastic adherence |  | Adherence to plug | |
| Yau et al. (30) | Karyotype, tumorigenicity, sterility, endotoxins, mycoplasma; screen patients for anti-HLA antibodies* | STRO-1, CC-9, HLA class I and II* |  | Yes* |  |
| Levy et al. (31) | Endotoxin, Gram stain, qPCR for viruses | Morphology, CD105, CD73, CD90 |  | Yes  (Trypan blue) | ≥ 70%  (parent cell bank) |
| Singer et al. (32) | Sterility, mycoplasma and cytogenetic analysis | Morphology |  |  |  |
| Myerson et al. (33) |  |  |  |  |  |
| Schweizer et al. (34) |  | QC tests performed but not specified |  | Yes |  |
| Powell et al. (35) | Endotoxin | CD90+, CD73+, CD105+, CD166+, CD45-, CD14-, CD34- and HLA-DR- |  | Yes  (Trypan blue) | 80-95% |
| Chan et al. (36) | Pathogens including endotoxins | CD90+, CD44+, CXCR4-, CD34-, C-kit-, CD144-, CD54-, CD45- and CD31-  (tested after expansion and before administration) |  |  |  |
| Harris et al. (37) | Sterility, karyotype | Surface marker profiling, osteo and adipogenesis, morphology, growth | Potency, growth | Yes  (Trypan blue) |  |
| McIntyre et al. (38) | Sterility, adventitious agents and mycoplasma, genotyping stability | Surface marker profiling, morphology, growth | IDO-1 expression and T cell suppression | Yes  (Trypan blue) | ³ 80% |
| Matthay et al. (39) | Sterility, karyotyping (after cryo) | Surface marker profiling, multi-lineage differentiation |  | Yes  (Trypan blue) | ³ 70%  Measured 1h after prepration |
| Swaminathan et al. (40) | “Stability data”  (no further information) |  |  | Yes | 95%  (retained viability without aggregation) |
| Keller et al. (41) | Sterility, mycoplasma, endotoxin, residual FBS | Surface marker profiling, HLA identity | T cell inhibition and cytokine expression | Yes  (Trypan blue) | >88%  Diluted cells were sampled every hour to assess recovery, clumping, and viability |
| Tompkins et al. (42) | Aerobic and anerobic bacteria, fungus, mycoplasma and endotoxin | > 95% CD105+  (threshold > 80%)  ≤ 1% CD45+  (threshold < 2%) |  |  |  |
| Glassberg et al. (43) | Sterility, mycoplasma* | CD105, CD45, CFU-F* |  | Yes* |  |
| Dietz et al. (44) | Sterility, mycoplasma, cytogenetic stability | Surface marker profiling, plug cell count |  | Yes  (Trypan blue) | >95% |
| Golpanian et al. (45) |  |  |  | Yes |  |
| Florea et al. (46) | Gram stain, mycoplasma (PCR) and endotoxin ≤5 EU/mL | CD105+, CD45− |  | Yes | >70% |
| Saad et al. (47) | Sterility testing (anaerobic and aerobic culture) mycoplasma, endotoxin and karyotyping | Surface marker profiling |  |  |  |
| Butler et al. (48) |  | CD105+, CD73+, CD90+ |  |  |  |
| Bajestan et al. (49) | Sterility, endotoxin | Surface marker profiling, clonogenicity, multilineage potential |  | Yes  (at 4^o^C for 24 hours) | 80% |
| Hare et al. (50) |  |  |  | Yes | 80-90%  pre-administration |
| Harris et al. (51) | Sterility, karyotyping | Surface marker profiling, osteo- and adipogenesis, morphology, growth | Potency | Yes |  |
| Steinberg et al. (52) |  |  |  |  |  |
| Dhere et al. (53) | Sterility, endotoxin, mycoplasma and cytogenetic stability | Surface marker profiling | IDO-1 expression and PBMC proliferation  (4h activation with 10ng/ml IFN-γ) | Yes | >70% |
| Staff et al. (54) | Sterility, mycoplasma, cytogenetic stability | Surface marker profiling |  |  |  |
| Castillo-Cardiel et al. (55) |  |  |  | Yes | >90% |
| Coetzee et al. (56) |  |  |  |  |  |
| Patel et al. (57) |  | 5-55% CD90+,  45-95% CD45+* |  | Yes | >70% |
| Levy et al. (58) |  |  |  |  |  |
| Perin et al. (59) | Sterility, mycoplasma, endotoxin, karyotyping;  Patient safety: screen patients for antibodies against bovine, murine or HLA antigens | Surface marker profiling |  | Yes  (Trypan blue)* | >70% |
| Levy et al. (60) |  |  |  |  |  |
| Skyler et al. (61) | Sterility, mycoplasma, endotoxin, transmissible infectious diseases, karyotyping, tumorigenicity | STRO-1+, CC-9 (CD146)+, and HLA class I and II | T-cell proliferation | Yes | >70% |
| Wilson et al. (62) | Sterility, karyotyping | Surface marker profiling, multi-lineage differentiation |  | Yes  (Trypan blue) | 50-63% |
| Maziarz et al. (63) | Bacterial and fungal contamination, chromosomal stability |  |  | Yes |  |
| Pettine et al. (64) |  | CD2+, CD3+, CD8+, and CD11b+, CD34+, CD90+, and CD105+  CFU-F and CFU-O |  | Yes (total nucleated cell count) | 98% |
